# Supplementary material for: Age-related immune response heterogeneity to SARS-CoV-2 vaccine BNT162b2
Source: Nature. 2021 Jun 30;596(7872):417–22. doi: 10.1038/s41586-021-03739-1 (PMC8373615; doi:10.1038/s41586-021-03739-1)
Supplement: Supplementary file 1 — Reporting Summary [file 41586_2021_3739_MOESM1_ESM.pdf]

nature research

Corresponding author(s): DBPR: 2021-04-05997D

Last updated by author(s): Jun 15, 2021

Reporting Summary

Nature Research wishes to improve the reproducibility of the work that we publish. This form provides structure for consistency and transparency in reporting. For further information on Nature Research policies, see Authors & Referees and the Editorial Policy Checklist.

Statistics

For all statistical analyses, confirm that the following items are present in the figure legend, table legend, main text, or Methods section.

n/a	Confirmed	
☐	☒	The exact sample size (*n*) for each experimental group/condition, given as a discrete number and unit of measurement
☐	☒	A statement on whether measurements were taken from distinct samples or whether the same sample was measured repeatedly
☐	☒	The statistical test(s) used AND whether they are one- or two-sided<br>*Only common tests should be described solely by name; describe more complex techniques in the Methods section.*
☐	☒	A description of all covariates tested
☐	☒	A description of any assumptions or corrections, such as tests of normality and adjustment for multiple comparisons
☐	☒	A full description of the statistical parameters including central tendency (e.g. means) or other basic estimates (e.g. regression coefficient) AND variation (e.g. standard deviation) or associated estimates of uncertainty (e.g. confidence intervals)
☐	☒	For null hypothesis testing, the test statistic (e.g. *F*, *t*, *r*) with confidence intervals, effect sizes, degrees of freedom and *P* value noted<br>*Give P values as exact values whenever suitable.*
☒	☐	For Bayesian analysis, information on the choice of priors and Markov chain Monte Carlo settings
☒	☐	For hierarchical and complex designs, identification of the appropriate level for tests and full reporting of outcomes
☐	☒	Estimates of effect sizes (e.g. Cohen's *d*, Pearson's *r*), indicating how they were calculated

Our web collection on statistics for biologists contains articles on many of the points above.

Software and code

Policy information about availability of computer code

Data collection

Graphpad Prism v9 was used to produce figures.
Stata v13 was used for statistical analyses.
FlowJo version 10.7.1 for flow cytometry analyses.
IMGT-V QUEST was used for immunoglobulin gene use and sequence annotation
B cell receptor repertoire analyses was performed in Python.

Data analysis

Logistic regression was used to model the association between age group and neutralisation by vaccine-elicited antibodies after the first dose of the BNT162b2 vaccine. The effect of sex and time interval from vaccination to sampling as confounders were adjusted for. Linear regression was also used to explore the association between age as a continuous variable and log transformed ID50, binding antibody levels, antibody subclass levels and T cell response after dose 1 and dose 2 of the BNT162b2 vaccine.

The difference in continuous and categorical data were tested using Wilcoxon rank sum or Mann-Whitney test and Chi square test respectively.

For manuscripts utilizing custom algorithms or software that are central to the research but not yet described in published literature, software must be made available to editors/reviewers. We strongly encourage code deposition in a community repository (e.g. GitHub). See the Nature Research guidelines for submitting code & software for further information.

Data

Policy information about availability of data

All manuscripts must include a data availability statement. This statement should provide the following information, where applicable:

- Accession codes, unique identifiers, or web links for publicly available datasets
- A list of figures that have associated raw data
- A description of any restrictions on data availability

Sequence data have been deposited at the European Genome -phenome Archive (EGA) which is hosted by the EBI and the CRG under accession number EGAS00001005380.

Field-specific reporting

Please select the one below that is the best fit for your research. If you are not sure, read the appropriate sections before making your selection.

☒ Life sciences ☐ Behavioural & social sciences ☐ Ecological, evolutionary & environmental sciences

For a reference copy of the document with all sections, see nature.com/documents/nr-reporting-summary-flat.pdf

Life sciences study design

All studies must disclose on these points even when the disclosure is negative.

Sample size	We assumed a risk ratio of non-neutralisation in the ≥80 years group compared with <80 years group of 5. Using an alpha of 0.05 and power of 90% required a sample size of 50 with a 1:1 ratio in each group. We however recruited 140 participants in order to reduce the risk of Type I error as multiple analyses were undertaken.
Data exclusions	Consecutively presenting participants were recruited with no exclusion.
Replication	Experiments were done in technical duplicates and a repeat was done.
Randomization	Not applicable as this was not an intervention study.
Blinding	Not applicable as this was not an intervention study.

Reporting for specific materials, systems and methods

We require information from authors about some types of materials, experimental systems and methods used in many studies. Here, indicate whether each material, system or method listed is relevant to your study. If you are not sure if a list item applies to your research, read the appropriate section before selecting a response.

Materials & experimental systems

n/a	Involved in the study	
☐	☒	Antibodies
☐	☒	Eukaryotic cell lines
☒	☐	Palaeontology
☒	☐	Animals and other organisms
☐	☒	Human research participants
☒	☐	Clinical data

Methods

n/a	Involved in the study	
☒	☐	ChIP-seq
☐	☒	Flow cytometry
☒	☐	MRI-based neuroimaging

Antibodies

Antibodies used	anti-CD4+ or anti-CD8+ direct beads (Miltenyi Biotec)<br>CD3-FITC, CD4-PE, and CD8- PerCPCy5.5 antibody (BioLegend)<br>CD19 (SJ25C, 363028), CD3 (OKT3, 317328), CD11c (3.9, 301608), CD25 (M-A251, 356126), CD14 (M5E2,301836), and IgM (IgG1-k, 314524) (all Biolegend).<br>CCR7 (150503, 561143) and IgG (G18-145, 561297) (BD Biosciences)<br>CD45RA (T6D11, 130-113-359) (Miltyeni Biotech)<br>CD8a (SK1, 48-0087-42) (eBiosciences)
Validation	Validation by manufacturer as detailed on their website.

Eukaryotic cell lines

Policy information about cell lines

Cell line source(s)	HEK 293T and HeLa cells were used.
Authentication	None of the cell lines used were authenticated.
Mycoplasma contamination	All cell lines used were tested (by PCR) and were mycoplasma free.
Commonly misidentified lines (See ICLAC register)	No commonly misidentified lines were used in this study.

Human research participants

Policy information about studies involving human research participants

Population characteristics	Community participants or health care workers receiving the first dose of the BNT162b2 vaccine between the 14th of December 2020 to the 10th of February 2021 were consecutively recruited. 140 participants were recruited.
Recruitment	Participants attending Addenbrooke's Hospital for their COVID-19 vaccination were recruited through the NIHR BioResource Centre Cambridge.
Ethics oversight	The study was approved by the East of England – Cambridge Central Research Ethics Committee.

Note that full information on the approval of the study protocol must also be provided in the manuscript.

Flow Cytometry

Plots

Confirm that:

☒ The axis labels state the marker and fluorochrome used (e.g. CD4-FITC).

☒ The axis scales are clearly visible. Include numbers along axes only for bottom left plot of group (a 'group' is an analysis of identical markers).

☒ All plots are contour plots with outliers or pseudocolor plots.

☒ A numerical value for number of cells or percentage (with statistics) is provided.

Methodology

Sample preparation	PBMCs were isolated from whole blood samples using Lymphoprep and stored in liquid nitrogen. Cells were thawed and stained in PBS containing 2nM EDTA at 4 °C
Instrument	Instrument: FACSAria Fusion (BD Biosciences).
Software	FlowJo version 10.7.1
Cell population abundance	2 x 10^5 to 2 x 10^6
Gating strategy	Total lymphocytes were gated, then live dead. From live dead, CD3- and CD3+ cells were separated. CD3+ cells were separated into CD8- and CD8+ cells. CD8+ memory cells were determined using CCR7 and CD45RA from the CD8+ gate. From the CD8- gate, CD4 memory cells were determined using CCR7 and CD45RA. From the CD4 memory gate, Tregs were separated from CD4 memory cells with CD25. The CD3- gate was used to separate CD14- and CD14+ cells. CD14- cells were separated in CD19+CD56- and CD19-CD56+ gates. From the CD19+ gate, B memory cells were determined using IgG and IgM. IgG+IgM- cells were gated and from these cells, B memory cells doubly positive for Spike were gated.

☒ Tick this box to confirm that a figure exemplifying the gating strategy is provided in the Supplementary Information.
